# Active, Passive, and Non-Sexting Adolescents: Testing Deviancy and Normalcy Perspectives Across Risk-Related and Sexual Competence Variables

**DOI:** 10.1007/s10508-026-03477-3

**Published:** 2026-07-01

**Authors:** Katharina Brand, Sascha Hein, Barbara Krahé, Paulina Tomaszewska, Isabell Schuster

**Affiliations:** 1 Department of Education and Psychology, Freie Universität Berlin, Fabeckstraße 35, 14195 Berlin, Germany; 2 Child Study Center, Yale University, New Haven, CT USA; 3 Department of Psychology, University of Potsdam, Potsdam, Germany; 4 Department of Anesthesiology and Intensive Care Medicine - Interdisciplinary Multimodal Pain Therapy, Charité Universitätsmedizin, Berlin, Germany

**Keywords:** Adolescents, Sexting, Risky sexual behavior, Depression, Sexual competence

## Abstract

Two contrasting frameworks have guided research on adolescent sexting. The deviancy perspective regards sexting as a risky behavior. The normalcy perspective acknowledges consensual sexting as a form of sexual expression with potential benefits. Drawing on both perspectives, this study compared adolescents who actively engaged in voluntary sexting (i.e., sent a sext of themselves or others) with those who were only passively (i.e., received, asked, or were asked for a sext, but did not send one) or never involved in sexting. The three groups were compared regarding sexuality-related cognitions, sexual behavior, and depressive symptoms, considering risk- and competence-related variables of sexual development. A sample of 216 adolescents (*M*_age_ = 16.93) completed an online questionnaire. Half reported active sexting (AS; active sexters), 16.2% exclusively passive sexting (PS; exclusively passive sexters), and 33.8% no sexting (NS; non-sexters). Supporting the deviancy perspective, AS reported higher levels on all risk-related variables, including risky sexual behavior, risky sexual scripts, acceptance of sexual coercion, perceived realism/utility of pornography, non-violent and violent pornography use, and depressive symptoms, than NS. Several significant differences emerged between AS and PS. Contrary to our hypotheses informed by the normalcy perspective, no significant group differences were found in indicators of sexual competence, namely communication about sexuality, sexual self-esteem, and sexual assertiveness. Results suggest that active sexting is embedded in a broader constellation of risk-related sexual behaviors and cognitions. Potential benefits of sexting may depend on factors not captured in the study, such as relationship context, underscoring the need for future research.

**Supplementary Information:**

The online version contains supplementary material available at 10.1007/s10508-026-03477-3.

## Introduction

Adolescence is a developmental period crucially characterized by increasing sexual curiosity and exploration (Kar et al., 2015). The evolution of digital media introduces new behaviors and possibilities into adolescents’ sexual repertoires (e.g., Maes et al., 2022). For most adolescents, accessing digital devices such as smartphones, social media, and virtual communication has become an integral part of their daily lives (Faverio & Sidoti, 2024). In this online environment, sexting has emerged as a contemporary form of sexual expression (Pistoni et al., 2023), attracting academic interest regarding its potential consequences and the characteristics of adolescents who engage in it. Sexting has become part of “mainstream culture” (Mori et al., 2019). Studies involving samples with a mean age under 18 years indicate that around 15% to 35% of adolescents engage in sexting behaviors (see Mori et al., 2022, for a meta-analysis). Many studies have framed sexting from a deviancy perspective, considering the behavior as a risky practice (Kosenko et al., 2017). Less attention has been given to its manifold role in sexual development during adolescence (Döring, 2014), let alone its potential benefits (Van Dijck et al., 2025). A balanced perspective, considering potential risks alongside developmental functions of sexting, may help to capture the complexity of adolescent sexting and inform sexual education efforts. Therefore, the present study aims to contribute to a nuanced understanding of sexting by examining differences in sexuality-related cognitions, sexual behaviors, and depressive symptoms among adolescents voluntarily engaging in active sexting (i.e., those who have sent a sext of themselves or others), exclusively passive sexting (i.e., those who have received a sext, have asked for a sext, or have been asked for a sext, but have not sent one), and non-sexting adolescents (i.e., those without active or passive sexting involvement), following the differentiation proposed by Barrense-Dias et al. (2017). By contrasting these forms of sexting, we take potential benefits into account and move beyond the deviancy perspective that regards sexting as a risky behavior only.

### Definition and Prevalence of Sexting

Sexting is a portmanteau of “sex” and “texting.” It can be defined as the exchange of sexually explicit messages, images, or videos through digital means (Madigan et al., 2018), commonly referred to as sexts (Döring, 2014). Despite a growing body of research, there is no consensus on the research paradigm for studying sexting. Studies have focused on different aspects, such as the specific action involved (e.g., sending, receiving, forwarding, or requesting), the types of media exchanged (e.g., text messages, images, or videos), the explicitness of the content (e.g., sexually explicit, sexy, or nude images), and the presence of consent (see Barrense-Dias et al., 2017, for a review). Due to this variety of aspects, the prevalence of sexting among adolescents varies considerably across studies. In a meta-analysis of 28 studies conducted between 2016 and 2020, including 48,024 participants with a mean age of 14.9 years, Mori et al. (2022) estimated that 19.3% of adolescents sent and 34.8% received a sext, and 14.5% forwarded a sext without the sender’s consent. A systematic review by Barrense-Dias et al. (2017) distinguished between passive sexting (i.e., receiving, asking for, or being asked for sexts) and active sexting (i.e., sending or forwarding sexts). Prevalence rates for passive sexting among adolescents under 18 years ranged from 7.6% to 60.0%, while prevalence rates for active sexting ranged from 0.9% to 27.6%. These rates must be considered in relation to different time windows, research methods, and samples in the respective studies.

### Demographic Correlates of Sexting

Beyond prevalence rates, studies have examined sociodemographic characteristics that correlate with adolescents’ involvement in sexting. Studies report inconsistent findings on gender differences in sexting. Some studies estimated similar rates of sending sexually explicit content among male and female adolescents (Gámez-Guadix & de Santisteban, 2018), while others suggested that female adolescents are more likely to send sexts (Ybarra & Mitchell, 2014) or, conversely, that male adolescents are more likely to send sexts (Morelli et al., 2017).

A more consistent pattern emerges with respect to age. Several studies indicate that the likelihood of sexting increases as adolescents get older (e.g., Mori et al., 2022). In the study by Gámez-Guadix and de Santisteban (2018), 2.8% of the 12-year-old participants had voluntarily sent a sext in the past year, compared to 31.1% of the 16-year-old participants. Madigan et al. (2018) explained the increasing prevalence of sexting with age through the growing availability and ownership of smartphones, as well as relational and sexual development throughout adolescence. Indeed, adolescents who engage in sexting are more likely to be sexually experienced (Kosenko et al., 2017; Mori et al., 2019). In their meta-analysis, Handschuh et al. (2019) pooled data from 9676 adolescents and found that the odds of reporting sexual activity were 6.3 times higher (95% CI, 4.9–8.1) for adolescents who had sent a sext compared to their non-sexting peers. A longitudinal study by Temple and Choi (2014) found that sending a sext (i.e., active sexting) was positively associated with having sex in the next year, while passive sexting behaviors (i.e., asking or being asked for a sext) showed no direct, but an indirect association with having sexual intercourse in the next year via active sexting.

Sexual orientation has also been identified as a significant correlate of adolescent sexting behavior. A systematic review by Barroso et al. (2023) identified five studies that found an association between sexual orientation and sexting. Specifically, sexual minority adolescents were more likely to engage in consensual sexting than their heterosexual peers. For example, Van Ouytsel et al. (2021) found that 25.0% of lesbian, gay, or bisexual adolescents had sent a sexually explicit image in the two months preceding the study, compared to 8.7% of heterosexual respondents. Because non-heterosexual adolescents often encounter limited opportunities and potential stigma in offline settings, they may be more likely to use digital communication as a space to seek intimacy and explore their sexual identity (Pinsky, 2022).

Most of the available literature has documented negative associations and consequences of sexting among adolescents. However, some authors have argued that sexting may also play a potentially beneficial role in the sexual development of adolescents, but this perspective has received less attention (Kosenko et al., 2017; Van Dijck et al., 2025). We will summarize the two contrasting perspectives on sexting before introducing the aims of the present study.

### Sexting: The Deviancy Perspective

In public and academic discourse, adolescent sexting is predominantly framed from a deviancy perspective, portraying it as a problematic behavior and a troubling development in adolescents’ use of digital media (Döring, 2014). A primary concern is the loss of control over shared content, particularly sexual images or videos. Once an image or video is shared, it can be distributed by the initial recipient without the sender’s consent (Martínez Soto et al., 2024). Such unauthorized distribution can lead to humiliation, harassment, or bullying of the victim (Barrense-Dias et al., 2017). Additionally, these images can be exploited for ‘sextortion,’ where individuals are coerced through threats of exposure (Van Ouytsel et al., 2021). Consequently, the deviancy perspective regards sexting as a risky practice (Döring, 2014), with a considerable number of studies focusing on its associations with risky sexual behavior and health-related risk behaviors. A meta-analysis by Mori et al. (2019) encompassing 23 studies with a total of 41,723 participants under the age of 18 years found that adolescent sexting was associated with higher odds of alcohol use, drug use, smoking, and delinquent behavior. Sexting adolescents were also more likely to engage in risky sexual behavior, such as unprotected sex and having multiple sexual partners. Further studies have linked sexting to sexual activity after consuming alcohol or using substances (Benotsch et al., 2013), experience of sexual coercion for female adolescents (Choi et al., 2016), and perpetration of sexual coercion by male adolescents (Stanley et al., 2018). In their cross-sectional study, Titchen et al. (2019) examined adverse life experiences and their associations with sexting among adolescents. For female participants, being a victim of physical or sexual intimate partner violence (IPV) was associated with the sending of sexts, whereas IPV perpetration showed no association. In contrast, for male participants, IPV perpetration was associated with sexting, while IPV victimization showed no significant association. Taken together, sexting adolescents are more likely to engage in risky (sexual) behavior and are more likely to experience (female participants only) or perpetrate (male participants only) sexual coercion.

Building on past findings presented above, it can be assumed that differences also emerge in sexuality-related cognitions. A central theory that has been associated with risky sexual behavior and sexual coercion is the sexual script theory (e.g., Krahé et al., 2022). Sexual scripts can be described as cognitive representations of typical elements of consensual sexual interactions that guide sexual behavior (Simon & Gagnon, 1986). They contain descriptive elements, specifying which typical behaviors and features are encountered in a sexual interaction, and normative elements, representing beliefs about appropriate conduct in sexual interactions. If sexual scripts comprise elements shown to increase the odds of sexual coercion (e.g., alcohol consumption in sexual situations, casual sexual partners), they can be considered “risky” (Krahé et al., 2007). Thus, adolescents who engage in sexting may be more likely to hold riskier sexual scripts related to sexual aggression than their non-sexting peers.

Furthermore, research has linked sexting and pornography use (e.g., Clancy et al., 2021). A recent cross-sectional study by Feijoo et al. (2025) found a greater participation of adolescents who consumed pornography in several online and offline risk behaviors, including sexting, compared to those who did not use pornography. Clancy et al. (2021) proposed that exposure to pornography may shape cognitive scripts about self-representation and sexual behavior that are subsequently enacted through sexting. Moreover, pornography consumption is linked to its perceived realism, which appears to be an important factor in the influence of pornography on sexual scripts (Gunnoo & Powell, 2023; Krahé et al., 2022). It remains unclear whether sexting adolescents differ in their perceptions of pornography realism and utility, as this aspect has yet to be assessed. Research on adolescents’ use of pornography should also differentiate between types of pornographic content (Raine et al., 2020). For instance, a cross-sectional study by Romito and Beltramini (2015) with Italian adolescents found that taking sexual photos was associated with increased odds of exposure to violent or degrading pornography. Among female adolescents, taking sexual photos was associated with five times higher adjusted odds (95% CI, 1.89–13.17) of exposure to violent or degrading pornography compared to no pornography exposure. Among males, the adjusted odds were 2.4 times higher (95% CI, 1.06–5.42) relative to exposure to non-violent pornography only.

Additionally, associations between sexting and psychopathology, such as depression and anxiety, have been documented. A systematic review by Gassó et al. (2019) suggested a potentially bidirectional relationship between sexting and depressive symptoms and noted higher levels of anxiety in sexting adolescents. Similarly, the meta-analysis by Mori et al. (2019) reported that adolescents who sext are more likely to experience depressive and anxiety symptoms than their non-sexting peers, with this association being particularly pronounced among younger adolescents. However, other studies have not found an association between sexting and depression or anxiety symptoms (e.g., Morelli et al., 2016).

Kosenko et al. (2017) proposed that the problem-behavior theory (PBT; see Jessor, 2001) offers a theoretical framework for understanding the link between sexting and risk behaviors among adolescents, as emphasized by the deviancy perspective. PBT conceptualizes adolescent problem behaviors as actions that are socially disapproved (e.g., aggression) and has been extended to include health-compromising behaviors and mental health difficulties (Jessor, 2001). According to the PBT, problem behaviors share common underlying causes in personality, environmental, and behavioral systems. Consequently, adolescents who engage in one type of problem behavior (e.g., sexting) are more prone to engage in other problem behaviors (e.g., substance use).

### Sexting: The Normalcy Perspective and Potential Benefits

A growing normalcy discourse challenges the risk-oriented deviancy perspective around sexting that has dominated past research. This perspective frames sexting, when done consensually, as a “contemporary form of sexual expression and intimate communication in romantic and sexual relationships” (Döring, 2014, p. 6). Rather than viewing sexting solely through the lens of risk and deviancy, this approach recognizes sexting as a mechanism for individuals to explore, develop, and express their sexuality as well as to initiate and maintain intimate relationships (Cooper et al., 2016; Döring, 2014). Thus, the sending of a sext may reflect and correspond to the sexual developmental needs of adolescents (Maes et al., 2022).

The deviancy perspective tends to disregard the motivations and aspects of sexting perceived by the adolescents involved (Cooper et al., 2016). However, sexual purposes, such as feeling sexually aroused or increasing passion and intimacy within a relationship, may serve as the primary motive for sexting adolescents (Bianchi et al., 2016). This was followed by motivations related to body reinforcement (e.g., to test one’s attractiveness; Bianchi et al., 2016). Regarding potential consequences of sexting, a study among 469 undergraduate students found that 79.3% of sexting participants retrospectively perceived their overall sexting experience as positive, while 20.7% considered it to be negative (Hudson & Marshall, 2018). Participants indicated several positive consequences of sexting, including sexual arousal, a greater acceptance of their bodies, and improvements in their relationship and communication (e.g., being able to communicate feelings). In their scoping review of the literature, Van Dijck et al. (2025) identified three main and potentially overlapping benefits associated with sexting. First, individual benefits may arise from the act itself, such as sexual pleasure and sexual exploration. Second, individual benefits may result from the reaction of the sexting partner, including feelings of validation and being desired. Lastly, Van Dijck et al. emphasized relational benefits, such as increased sexual satisfaction or enhanced trust through self-disclosure within the relationship. Van Dijck et al. called for more research addressing the potential benefits of sexting, specifically the benefits of consensual sexting, as research examining sexting through a sex-positive lens remains limited. Pointing to the potential relevance of sexting in the sexual development of adolescence, Marengo et al. (2019) found that adolescents who sent a sext had a higher sexual self-concept, conceptualized as a positive attitude towards sex and confidence in one’s own sexuality, and a higher sex drive than their non-sexting peers.

Given that adolescence is a period of sexual exploration and development (Kar et al., 2015), the potential benefits of sexting should be considered beyond the traditional risk-focused discourse (Van Dijck et al., 2025). An important aspect of adolescents’ sexual development is the gaining of sexual competence, which refers to the acquisition of skills necessary to attain pleasurable and safe sexual experiences (e.g., Hirst, 2008). Sexual competence includes assessing one’s sexual self (i.e., sexual self-esteem), communicating sexually with a partner (e.g., about sexual preferences and dislikes), and demonstrating sexual assertiveness (Tomaszewska et al., 2022). Sexual assertiveness is embedded in sexual interactions and is expressed by refusing unwanted sexual interactions (i.e., refusal assertiveness) and initiating wanted sexual activity (i.e., initiation assertiveness; Tomaszewska et al., 2022). Comparing sexting adolescents to their non-sexting peers on these aspects of sexual competence will provide valuable insights into the broader developmental implications of sexting behavior.

### The Current Study

Building on the presented definition of sexting as the exchange of sexually explicit content through digital means (Madigan et al., 2018), the current study assessed the consensual digital exchange of sexually explicit images. Following Barrense-Dias et al. (2017), sexting behaviors were categorized as either active, which involves the sending of sexually explicit images of oneself or the forwarding to others, or passive, referring to receiving such content, being asked to send sexts, or asking others to do so. Although asking for a sext involves an active behavioral component, it does not entail the direct production or distribution of sexually explicit content (e.g., messages, images, or videos), which are central to active sexting (Barrense-Dias et al., 2017). Thus, passive sexting engagement reflects a different level of involvement in the sexting exchange and is therefore conceptually different from active sexting.

Many studies have focused on active sexting behaviors (e.g., Bianchi et al., 2016; Marengo et al., 2019; Ybarra & Mitchell, 2014) as they directly involve the sending of sexually explicit content, thereby posing potential risks for the involved individual (e.g., further distribution). However, as indicated by the normalcy perspective, the sending of a sext may also be associated with developmental benefits, as it allows individuals to express and explore their sexuality. Therefore, when compared to adolescents who are exclusively passively involved in sexting, adolescents who decide to actively send sexts may be at greater risk, but they may also experience greater developmental benefits.

The study aimed to expand the existing literature on adolescent sexting by moving beyond the predominant deviancy perspective and incorporating a more comprehensive developmental lens. Acknowledging both the deviancy and normalcy perspectives on adolescent sexting, this study examined differences between sexting adolescents, divided into active and passive sexting groups, and non-sexting adolescents across sexuality-related cognitions, sexual behaviors, and depression. Specifically, we investigated variables that align with the deviancy perspective (i.e., risky sexual behavior, violent and non-violent pornography use, perceived realism and utility of pornography, acceptance of sexual coercion, and depression) and those that align with the normalcy perspective (i.e., sexual self-esteem, communication about sexuality, and sexual assertiveness), acknowledging potential benefits. By integrating these perspectives, the study aimed to provide a more nuanced understanding of sexting. The findings contribute to ongoing discussions about adolescent digital sexual expression, balancing risk awareness with the recognition of sexting as a potential benefit of sexual development. Furthermore, we examined differences between the sexting groups in terms of gender, age, sexual orientation, and sexual experience, as previous studies have identified these demographic and sexual characteristics as correlates of sexting. However, since the study focuses on variables related to perspectives of normalcy and deviancy, we investigated the differences in an exploratory manner, without stating specific hypotheses.

### Hypotheses Informed by the Deviancy Perspective

Based on the deviancy perspective, the following hypotheses were tested:

#### Hypothesis 1:

Adolescents who actively engage in sexting exhibit riskier sexual behaviors than their non-sexting peers or those who only engage in passive sexting.

#### Hypothesis 2:

Adolescents who actively engage in sexting report more risky sexual scripts and a higher acceptance of sexual coercion than their non-sexting peers or those who only engage in passive sexting.

#### Hypothesis 3:

Adolescents who actively engage in sexting report higher frequencies of non-violent and violent pornography use, as well as greater perceived realism and utility of pornography than their non-sexting peers or those who only engage in passive sexting.

#### Hypothesis 4:

Adolescents who actively engage in sexting exhibit higher levels of depression compared to their non-sexting peers or those who only engage in passive sexting.

### Hypotheses Informed by the Normalcy Perspective

Aligned with the normalcy perspective, the study included sexuality-related cognitions and sexual behaviors that can be regarded as indicators of sexual competence. The following hypotheses were tested:

#### Hypothesis 5:

Adolescents who actively engage in sexting exhibit higher sexual self-esteem than their non-sexting peers or those who only engage in passive sexting.

#### Hypothesis 6:

Adolescents who actively engage in sexting report more communication about sexuality than their non-sexting peers or those who only engage in passive sexting.

#### Hypothesis 7:

Adolescents who actively engage in sexting report higher refusal and initiation assertiveness compared to their non-sexting peers or those who only engage in passive sexting.

## Method

### Participants

We collected data online during the summer and fall of 2021 under the study title ‘Adolescents and Sexuality.’ Recruitment of participants involved distributing flyers and emails with the study link to youth centers and organizations and sharing the study information on social media platforms. A total of 228 participants completed the online survey. We excluded participants younger than 15 and older than 18 (*n* = 2). Additionally, we excluded participants who completed the survey but reported their gender as diverse (*n* = 5), as their small group size did not allow stratified analyses. We also excluded participants who did not answer the sexting behavior items (*n* = 5). The final sample consisted of 216 adolescents (female: *n* = 150, 69.4%; male: *n* = 66, 30.6%) aged between 15 and 18 years. The mean age was 16.9 years (*SD* = 0.93), with no significant difference in age between female and male adolescents (*t*(214) = -0.94, *p* = .349). Most of the sample (94.9%) held German nationality, either exclusively or combined with another nationality. Regarding educational aspirations, more than half of the participants aimed for higher secondary education (52.8%; Abitur), followed by intermediate secondary education (13.0%; Mittlerer Schulabschluss), lower secondary education (12.5%; Hauptschulabschluss), university degree (9.3%), vocational training (6.9%), and technical college qualification (5.1%; Fachhochschulreife). One participant (0.5%) did not disclose their educational aspirations. More than half of the participants (59.3%; *n* = 128) identified as exclusively heterosexual, while 39.3% (*n* = 85) identified as non-exclusively heterosexual. Three participants (1.4%) did not state their sexual orientation. Furthermore, 82.9% (*n* = 179) of participants reported sexual experiences with a partner, including activities such as kissing, intimate touching, or sexual intercourse, whereas 17.1% (*n* = 37) reported no partnered sexual experience.

### Measures

#### Sexting

The study adapted four items from Van Ouytsel et al. (2019) to capture participants’ engagement in sexting behaviors. We added to the original items that sexting behaviors were voluntary, extended the wording so that all items referred to sexually explicit images of oneself or others, and changed the reference period from the past six months to lifetime. Three items addressed passive sexting experiences, such as being asked for, requesting, or receiving sexually explicit images (i.e., “Has anyone ever asked you to send sexually explicit photos of yourself or others via mobile phone or computer, which was ok for you?”; “Have you ever asked someone to send sexually explicit photos of themselves or others via mobile phone or computer”; and “Has anyone ever sent you sexually explicit photos of themselves or others via mobile phone or computer, which you received voluntarily?”).^1^ One item focused on active sexting (“Have you ever voluntarily sent sexually explicit photos (e.g., nude photos) of yourself or others via mobile phone or computer?”).^1^ Participants rated the frequency of these behaviors on a five-point scale ranging from 1 (*never*) to 5 (*very often*). A mean score across the three passive sexting items was computed to indicate the overall frequency of voluntary engagement. The subscale demonstrated good internal consistency (Cronbach’s α = .77).

#### Measures Aligned with the Deviancy Perspective

**Risky Sexual Behavior.** To measure risky sexual behavior, participants with prior sexual experience completed nine items from previous research (Schuster et al., 2023). We further added three items on the ambiguous communication of sexual intention. Participants reported the frequency of engaging in specific behaviors during sexual encounters on a five-point scale ranging from 1 (*never*) to 5 (*very often*). The items addressed three dimensions: (1) sexual contact with casual partners (three items; e.g., “How often have you had sexual contact with a girl/boy on your first date?”); (2) alcohol use during sexual contact (four items; e.g., “How often did you drink alcohol in situations in which you had sexual contact?”); and (3) ambiguous communication of sexual intentions (five items; e.g., “In situations in which you had sexual contact, how often have you talked about whether you want to be intimate with each other?”; reverse-coded). Averaging responses across all twelve items provided a total score, with higher scores indicating more frequent risky sexual behavior. Due to our adaptation of the measure, direct comparability with previous research is limited. Internal consistency was good (Cronbach’s α = .78).

**Risky Sexual Scripts.** Following previous studies with young adults (Schuster et al., 2022; Tomaszewska & Schuster, 2019), we used a composite measure to assess participants’ risky scripts for consensual sexual encounters, differentiating between descriptive and normative features. The two components were initially developed for adolescents and have demonstrated good internal consistencies in previous research (Krahé et al., 2007). The first component measured the descriptive content of risky sexual scripts. Participants were asked to imagine a scenario in which they have sexual intercourse with a new partner and to rate how likely the following elements would be present in this situation: (1) engaging in casual sexual contact and length of acquaintanceship (three items; e.g., “How likely is it that you would have known each other before?”); (2) consumption of alcohol and degree of intoxication (four items, e.g., “How likely is it that you drink alcohol in that situation?”); and (3) ambiguous communication of sexual intentions (five items; e.g., “How likely is it that you consent to sexual acts that you don't actually want?”). Response options for casual sex, alcohol use, and ambiguous communication of sexual intentions ranged from 1 (*very unlikely*) to 5 (*very likely*). Level of intoxication was rated from 1 (*not at all*) to 5 (*totally*), and length of acquaintanceship from 1 (*not at all*) to 5 (*a few months or longer*). The descriptive scale demonstrated acceptable internal consistency (Cronbach’s α = .64).

The second component addressed the normative endorsement of risky sexual script elements with eleven items across the same three categories: (1) engagement in casual sex (two items; e.g., “I would find it okay to have sex with somebody without having had a date before.”); (2) consumption of alcohol in sexual situations (four items; e.g., “I wouldn’t mind having sex with somebody who has had too much to drink.”); and (3) ambiguous communication of sexual intentions (five items; e.g., “In my opinion, it is part of sex to be clear about one’s sexual desires.”; reverse-coded). Participants rated their level of agreement on a five-point scale ranging from 1 (*do not agree at all*) to 5 (*completely agree*). The normative scale demonstrated acceptable internal consistency (Cronbach’s α = .64). To create an overall index of risky sexual scripts, we multiplied the mean scores of the descriptive and the normative components. The resulting total score ranged from 1 to 25, with higher scores reflecting riskier sexual scripts.

**Acceptance of Sexual Coercion.** To assess the extent to which participants find the use of sexual coercion acceptable, we used seven items from previous studies (Krahé et al., 2007; Schuster et al., 2022). Participants were asked to imagine a scenario where one person wants to have sex with another who explicitly says no. They then rated seven justifications for sexual coercion (e.g., “I would find it acceptable if they have already discussed sexual topics.”) on a five-point scale ranging from 1 (*absolutely not*) to 5 (*absolutely yes*). We averaged the responses across the seven justifications to calculate a total score, with higher scores indicating greater acceptance of sexual coercion. The measure demonstrated high internal consistency in the present sample (Cronbach’s α = .91), comparable to the reliability estimates reported in a longitudinal study among college students (Cronbach’s α = .86–.93; Schuster et al., 2022).

**Non-Violent and Violent Pornography Use.** Participants reported their intentional use of pornography in the past 12 months. Four items assessed non-violent pornography use (e.g., “Persons showed what they sexually wanted.”) and four items addressed violent pornography use (e.g., “Actions that are carried out even though a person protests.”). These items were also used by Schuster et al. (2025) with the same dataset. Participants rated their frequency of exposure to pornographic material with either non-violent or violent content elements on a six-point scale, ranging from 1 (*never*) to 6 (*daily*). Scores for non-violent and violent pornography use were averaged separately to provide an overall frequency score for each category. Both scales demonstrated good internal consistency (non-violent pornography use: Cronbach’s α = .93; violent pornography use: Cronbach’s α = .89).

**Perceived Realism and Utility of Pornography.** We adapted six items by Peter and Valkenburg (2010) to assess the perceived realism and utility of pornography. The original items refer to “sex on the Internet.” We adjusted the wording to “pornographic media.” Participants evaluated statements such as “Pornographic media provide valuable information about sex” and “Sex in pornographic media is similar to sex in real life” on a five-point scale, ranging from 1 (*do not agree at all*) to 5 (*totally agree*). The mean score across the six items was calculated to create an overall score of perceived realism and utility of pornography. The scale demonstrated good internal consistency (Cronbach’s α = .88), similar to previous research (Schuster et al., 2022).

**Depression.** We measured depressive symptoms with the abbreviated form of the Depression Screener for Teenagers (DesTeen-a; Allgaier et al., 2014; Pietsch et al., 2011). The DesTeen-a includes five items addressing key symptoms of depression within the last two weeks: (1) bad mood; (2) loss of energy; (3) diminished pleasure; (4) fatigability; and (5) sadness. Each item offers four response options reflecting increasing levels of symptom severity (e.g., I am almost always in a good mood “0”; I am often in a good mood “1”; I am rarely in a good mood “2”; I am never in a good mood “3”). The response scales was reversed for items 3, 4, and 5. We computed a sum score, with higher scores indicating greater depressive symptoms. The scale exhibited good internal consistency in the present sample (Cronbach’s α = .78). Two validation studies have shown excellent diagnostic accuracy of the DesTeen-a in adolescent samples (Allgaier et al., 2014; Pietsch et al., 2011).

#### Measures Aligned with the Normalcy Perspective

**Sexual Self-Esteem.** To measure sexual self-esteem, we used five items from the short version of the Sexual Self-Esteem Scale (Zeanah & Schwarz, 1996) and three items based on a sexual self-esteem measure by O’Sullivan et al. (2006). Participants rated the eight items (e.g., “I feel comfortable with my sexuality.”) on a five-point scale ranging from 1 (*do not agree at all*) to 5 (*totally agree*). Responses were averaged to compute the mean score, with higher scores indicating greater sexual self-esteem. The scale demonstrated acceptable internal consistency (Cronbach’s α = .71).

**Communication about Sexuality.** We used the communicative sexuality subscale of the Process-Based Consent Scale, developed and validated by Glace et al. (2021), to measure the extent of communication about sexuality with a sexual partner. In addition, we included one item that had loaded on the communicative sexuality factor in the author’s exploratory factor analysis, resulting in a seven-item measure. Participants with prior sexual experience rated statements such as “I tell my partner what I want sexually.” on a five-point scale ranging from 1 (*do not agree at all*) to 5 (*totally* agree). The mean score was calculated, with higher scores indicating more communication about sexuality. The measure demonstrated good internal consistency (Cronbach’s α = .85).

**Refusal and Initiation Assertiveness.** The abilities of sexually experienced participants to refuse unwanted sexual contact and initiate desired sexual contact were assessed with four items each from the Refusal and Initiation subscales of the Sexual Assertiveness Scale (SAS; Morokoff et al., 1997). The SAS was initially developed and validated in samples of women, demonstrating adequate test–retest reliability and construct validity (Morokoff et al., 1997). The scale has since been used in adolescent samples (Auslander et al., 2007) and mixed-gender samples, in which the Initiation and Refusal subscales showed good internal consistency (Cronbach’s α = .85 and .76, respectively; Granados et al., 2020). Example items are: “If a person wants to have sex with me, I give in, even if I don’t want to.” (reverse-coded; Refusal subscale); and “I get intimate with a person when I want to and when they also want to.” (Initiation subscale). Participants responded on a five-point scale ranging from 1 (*never*) to 5 (*always*). We calculated the mean scores for refusal and initiation assertiveness separately, with higher scores indicating a greater ability to refuse unwanted sexual advances or initiate sexual contact. The Refusal subscale showed acceptable internal consistency (Cronbach’s α = .62). Removing one item (“I wait for the person to touch my private areas instead of letting them know that’s what I want.”, reverse-coded) from the Initiation subscale improved reliability, increasing Cronbach’s α from .44 to .58.

#### Demographic Information and Sexual Experience Background

Participants were asked to indicate their gender (German: “Geschlecht”; female, male, diverse).^2^ Further, participants provided information about their age, nationality, and educational goals. To assess sexual experience, participants indicated whether they had engaged in sexual contact, such as kissing, intimate touching, or sexual intercourse, with (a) a male or (b) a female partner (*yes/no*). We broadly referred to past sexual contact without specifying whether the contact had occurred consensually. Further, we assessed participants’ sexual orientation using the seven-point scale by Kinsey et al. (1948), ranging from 1 (*heterosexual*) to 7 (*homosexual*).

### Procedure

The present study served as a pilot study for a longitudinal research project examining prevalence, predictors, and consequences of sexual aggression perpetration and victimization (pre-registration: https://osf.io/6qtg5/). Data collection was carried out online, and all measures were presented in German. Where no pre-tested German versions were available, the measures were translated into German using the back-translation method, in which the English original was translated into German and then back into English by bilingual speakers. The survey started with an information page outlining the explicit sexual content of the study. The information page emphasized that participation was voluntary and that participants could withdraw at any time without consequences. After providing informed consent, participants could access the survey questions. All participants first completed demographic questions and items assessing their sexual experience background. The survey included questions about sexting behaviors, sexuality-related cognitive constructs, depression, and pornography use for all participants, regardless of their sexual experience. Adolescents who reported sexual contact additionally completed measures assessing their behaviors and communication in sexual interactions. All participants received a gift voucher after completing the survey.

### Data Analysis

Data was analyzed using SPSS Statistics 29.0. For data preparation, gender was coded as “0” for female and “1” for male participants. Regarding sexual orientation, we followed previous studies (e.g., Morelli et al., 2016) and categorized participants into two groups according to their responses on the scale by Kinsey et al. (1948): (1) exclusively heterosexual (rating of 1, coded as “0”); and (2) non-exclusively heterosexual (ratings 2–7, coded as “1”). For sexual experience background, participants who disclosed no sexual contact with either gender were categorized as “not sexually experienced” (coded as “0”), whereas those reporting sexual contact with males, females, or both were classified as “sexually experienced” (coded as “1”).

To examine differences between adolescents with varying sexting engagement, we categorized participants into three groups based on their answers on the four sexting items: (1) non-sexters, who reported no involvement in either passive or active sexting; (2) exclusively passive sexters, who reported engagement in passive sexting behaviors at least rarely but had not sent a sexually explicit image; and (3) active sexters, who had sent a sexually explicit image of themselves or others at least rarely. Categorical measures of sexting behaviors are frequently used in sexting research (e.g., Clancy et al., 2021; Van Ouytsel et al., 2019), particularly when response frequencies are low or distributions are skewed. Because almost all participants who reported active sexting also reported passive sexting (97.2%), a fourth group of exclusively active sexters was not created, as their group size (*n* = 3) was too small for separate analyses. Descriptive analyses included the proportion of participants in the three sexting groups and their differences across gender, age, sexual orientation, and sexual experience background, as well as the means and correlations of the study variables.

Considering Huang’s (2019) critique of MANOVAs (which also applies to MANCOVAs), specifically their emphasis on multivariate effects rather than quantifying group differences on individual dependent variables, we followed the author’s recommendation and conducted separate linear regression analyses for each outcome. A Bonferroni-corrected alpha level of *p* < .005 (.05/11) was applied to account for multiple tests across 11 linear regression models. Each regression model included two dummy-coded grouping variables (see description below), and the covariates gender, age, sexual orientation, and, where applicable, sexual experience as predictors. As Huang (2019) emphasized, this approach allows the inclusion of covariates “without adding much complexity to the model” (p. 58), while maintaining interpretability.

Two dummy-coded grouping variables were created to test our hypotheses: one dummy variable identified non-sexters (NS; coded as 1); the other dummy variable identified exclusively passive sexters only (PS; coded as 1). Adolescents who actively engaged in sexting (active sexters, AS) served as the reference group and were coded as 0 on both dummy-coded grouping variables. This coding allowed for comparisons of the active sexting group with both the exclusively passive and the non-sexting groups. To examine differences between exclusively passive and non-sexting adolescents, we conducted an additional set of analyses using an alternative dummy-coding scheme in which non-sexters served as the reference group. The results are included in the supplementary material (Tables SM1-SM3). Frequency tables indicated that the proportion of missing data for the included variables was low (< 1.4% across all variables). Therefore, listwise deletion was applied separately for each regression model. The number of included cases for each outcome is reported in the corresponding regression results table.

A priori power analyses were conducted using G*Power 3.1 (Faul et al., 2007) to determine the required sample size for the planned regression models. The power analyses were based on models that included the two sexting dummy variables and the relevant covariates, resulting in six predictors for models examining sexuality-related cognitions, depression, and pornography use, and five predictors for sexual behavior outcomes, as sexual experience was not applicable because sexually inexperienced participants did not receive measures addressing sexual behavior. Power was set to 0.80, and a medium effect size was assumed (*f* ^2^ = 0.15). An alpha level of .005 was applied to account for multiple testing across the 11 models, which reduces statistical power and increases the required sample size. The required sample sizes (*N* = 142 for five predictors; *N* = 150 for six predictors) were exceeded in the present study.

## Results

### Descriptive Statistics

#### Sexting Groups and Differences in Covariates

The overall frequencies of sexting behaviors were low (passive sexting: *M* = 1.89, *SD* = 0.89; active sexting: *M* = 1.83, *SD* = 0.99). Overall, 33.8% (*n* = 73) of participants reported no engagement in sexting behaviors and were labeled as non-sexters (NS), 16.2% (*n* = 35) were categorized as exclusively passive sexters (PS), and 50.0% (*n* = 108) were classified as active sexters (AS). No significant gender differences were found in the assignment to the three groups (χ^2^(2) = 0.34, *p* = .844). Further, no significant differences were found in terms of age (*F*(2, 213) = 2.17, *p* = .116) or sexual orientation (χ^2^(2) = 1.04, *p* = .596). A significant difference was found between prior sexual experience and sexting group membership (χ^2^(2) = 26.57, *p* < .001). Analysis of standardized residuals indicated that the NS group included fewer participants with prior sexual experience than expected (adjusted standardized residual = − 5.15), whereas the AS group included more participants with prior sexual experience than expected (adjusted standardized residual = 3.79).

#### Overall Means of the Study Variables

Means, standard deviations, and sample sizes of the measured variables to be compared across the sexting groups are presented in Table 1, organized separately according to the deviancy and normalcy perspectives. Sexuality-related cognitions, depression, and pornography use were assessed in the total sample, while sexual behaviors could only be measured among participants with prior sexual experience.

**Table 1:** Means, standard deviations, and sample sizes of study variables aligned with the deviancy and normalcy perspectives

	Range	*M*	*SD*	*n*
*Variables aligned with the deviancy perspective*				
Risky sexual behavior	1–5	2.38	0.62	179
Risky sexual scripts	1–25	6.47	2.34	216
Acceptance of sexual coercion	1–5	2.07	0.96	216
Non-violent pornography use	1–6	2.73	1.30	216
Violent pornography use	1–6	1.81	1.11	216
Perceived realism/utility of pornography	1–5	2.09	0.79	215
Depression	0–15	4.85	2.94	216
*Variables aligned with the normalcy perspective*				
Sexual self-esteem	1–5	3.42	0.67	216
Communication about sexuality	1–5	3.62	0.82	178
Refusal assertiveness	1–5	3.32	0.82	178
Initiation assertiveness	1–5	3.29	0.88	178

Sample sizes were smaller for variables related to sexual behavior (i.e., risky sexual behavior, communication about sexuality, refusal and initiation assertiveness) because these scales were only presented to participants with prior sexual experience

#### Correlations between Study Variables and Covariates

Bivariate correlations of the study variables and covariates are presented in Table 2. Several significant correlations were observed between the study variables and gender, age, sexual orientation, and sexual experience. Male gender was significantly associated with more frequent violent and non-violent pornography use as well as higher sexual self-esteem. In contrast, female gender was significantly associated with greater depressive symptoms and higher refusal assertiveness. Older age was significantly linked to higher sexual self-esteem and more frequent violent and non-violent pornography use. Non-exclusively heterosexual sexual orientation was significantly associated with higher depressive symptoms, more communication about sexuality, higher refusal assertiveness, and lower acceptance of sexual coercion. Sexual experience was significantly associated with more risky sexual scripts.

**Table 2:** Bivariate correlations among study variables and covariates

Variables	1	2	3	4	5	6	7	8	9	10	11	12	13	14	15	16	17
1. Active sexting	–																
2. Passive sexting	.70***	–															
3. Gender	.02	.13	–														
4. Age	.03	− .06	.06	–													
5. Sexual orientation	.02	− .04	− .29***	.03	–												
6. Sexual experience	.22**	.30***	− .07	.03	− .02	–											
*Deviancy perspective variables*																	
7. Risky sexual behavior	.42***	.35***	− .10	.06	.14	.^c^	–										
8. Risky sexual scripts	.45***	.43***	.01	− .06	− .04	.22**	.74***	–									
9. Acceptance of sexual coercion	.32***	.34***	.11	− .00	− .21**	.07	.47***	.58***	–								
10. Non-violent pornography use	.25***	.25***	.50***	.18**	.01	.06	.24**	.27***	.24***	–							
11. Violent pornography use	.52***	.46***	.20**	.18**	− .08	.04	.51***	.48***	.46***	.54***	–						
12. Perceived realism/utility of pornography	.45***	.45***	.08	.02	− .08	.09	.54***	.57***	.58***	.34***	.65***	–					
13. Depression	.22***	.12	− .25***	− .12	.17*	.04	.26***	.30***	.20**	− .11	.11	.21**	–				
*Normalcy perspective variables*																	
14. Sexual self-esteem	.04	.03	.22***	.15*	− .12	.11	− .23**	− .25***	− .23***	.11	− .08	− .20**	− .40***	–			
15. Communication about sexuality	− .13	− .12	.03	.07	.16*	.^c^	− .26***	− .34***	− .36***	.13	− .14	− .37***	− .24**	.38***	–		
16. Refusal assertiveness	− .19*	− .27***	− .16*	.03	.22**	.^c^	− .31***	− .44***	− .44***	− .20**	− .42***	− .48***	− .05	.22**	.30***	–	
17. Initiation assertiveness	.11	.07	.06	.08	.01	.^c^	− .03	− .11	.05	.14	.06	− .01	− .25***	.36***	.38***	.25***	–

Gender coded as 0 = female, 1 = male. Sexual orientation coded as 0 = exclusively heterosexual, 1 = non-exclusively heterosexual. Sexual experience coded as 0 = not sexually experienced, 1 = sexually experienced. ^*c*^ Correlation could not be computed. * *p* < .05, ** *p* < .01, *** *p* < .001 (two-tailed)

Bivariate correlations between the study variables ranged from weak to strong (*r* = .20 to *r* = .74). The strongest associations were observed between risky sexual scripts and risky sexual behavior, and between violent pornography use and perceived realism/utility of pornography. All indicators of sexual competence were positively associated with each other. Associations ranged from *r* = .22 between sexual self-esteem and refusal assertiveness to *r* = .38 between communication about sexuality and initiation assertiveness, as well as sexual self-esteem.

### Hypotheses-Testing Analyses

To test our hypotheses regarding group differences, we conducted separate linear regressions for each dependent variable. This approach facilitated comparisons between AS and PS, as well as between AS and NS, while accounting for the potential associations with demographic and sexual characteristics. The dependent variables were regressed on the dummy-coded variables, controlling for gender, age, sexual orientation, and, where applicable, sexual experience. Starting with the hypotheses derived from the deviancy perspective, Hypothesis 1 was partially supported. As presented in Table 3, NS scored significantly lower on risky sexual behavior than AS, indicating that AS engaged in riskier sexual behavior. After applying the adjusted significance level, risky sexual behavior did not vary significantly between PS and AS.

**Table 3:** Results of linear regression analysis for risky sexual behavior as a variable aligned with the deviancy perspective, with sexting behavior (active sexters as reference group), gender, age, and sexual orientation as predictors

	Risky sexual behavior (*n* = 177)			
Predictor	*B (SE)*	95% CI [LL, UL]	β	*p*
Gender	− 0.15 (0.10)	[− 0.35, 0.04]	− .12	.127
Age	0.03 (0.05)	[− 0.06, 0.13]	.05	.472
Sexual orientation	0.09 (0.09)	[− 0.09, 0.28]	.08	.315
Passive sexters^a^	− 0.27 (0.12)	[− 0.50, -0.04]	− .17	.021
Non-sexters^a^	− 0.57 (0.10)	[− 0.77, -0.37]	− .41	< .001**

Reference groups: Gender (male = 1), sexual orientation (non-exclusively heterosexual = 1). ^a^The reference group comprises active sexters. A Bonferroni-corrected alpha level was applied (.05/11 = .005). * *p* < .005. ** *p* < .001

Table 4 summarizes the results of the linear regression analyses for sexuality-related cognitions, pornography use, and depressive symptoms in line with the deviancy perspective. Partially supporting Hypothesis 2, both NS and PS scored significantly lower on risky sexual scripts than AS. Furthermore, NS scored significantly lower on acceptance of sexual coercion compared to AS, while the comparison between PS and AS did not reach statistical significance. Consistent with Hypothesis 3, both NS and PS reported significantly less frequent violent pornography use than AS. Regarding non-violent pornography use, only NS reported significantly lower frequencies compared to AS. Additionally, both NS and PS reported significantly lower perceived realism and utility of pornography than AS. Providing partial support for Hypothesis 4, NS showed significantly lower sum scores on depression than AS. The comparison between PS and AS did not reach the adjusted level of statistical significance.

**Table 4:** Results of multiple linear regressions for sexuality-related cognitions, depression, and pornography use, aligned with the deviancy perspective, with sexting behavior (active sexters as reference group), gender, age, sexual experience, and sexual orientation as predictors

	Risky sexual scripts (*n* = 213)				Acceptance of sexual coercion (*n* = 213)				Non-violent pornography use (*n* = 213)			
Predictor	*B (SE)*	95% CI [LL, UL]	β	*p*	*B (SE)*	95% CI [LL, UL]	β	*p*	*B (SE)*	95% CI [LL, UL]	β	*p*
Gender	− 0.02 (0.33)	[− 0.67, 0.63]	− .00	.962	0.13 (0.14)	[− 0.15, 0.41]	.06	.356	1.47 (0.17)	[1.14, 1.80]	.52	< .001**
Age	− 0.17 (0.16)	[− 0.48, 0.14]	− .07	.282	− 0.01 (0.07)	[− 0.14, 0.13]	− .01	.920	0.19 (0.08)	[0.03, 0.35]	.14	.019
Sexual experience	0.44 (0.41)	[− 0.38, 1.25]	.07	.289	− 0.05 (0.18)	[− 0.40, 0.30]	− .02	.770	0.06 (0.21)	[− 0.36, 0.47]	.02	.784
Sexual orientation	− 0.27 (0.31)	[− 0.88, 0.34]	− .06	.390	− 0.38 (0.13)	[− 0.64, − 0.12]	− .20	.005	0.39 (0.16)	[0.08, 0.70]	.15	.014
Passive sexters^a^	− 1.33 (0.41)	[− 2.14, − 0.51]	− .21	.002*	− 0.51 (0.18)	[− 0.86, − 0.16]	− .20	.005	− 0.30 (0.21)	[− 0.71, 0.12]	− .09	.158
Non-sexters^a^	− 2.31 (0.34)	[− 2.99, − 1.63]	− .46	< .001**	− 0.57 (0.15)	[− 0.87, − 0.28]	− .28	< .001**	− 0.75 (0.18)	[− 1.09, − 0.40]	− .27	< .001**

	Violent pornography use (*n* = 213)				Perceived realism/utility of pornography (*n* = 213)				Depression (*n* = 213)			
Predictor	*B (SE)*	95% CI [LL, UL]	β	*p*	*B (SE)*	95% CI [LL, UL]	β	*p*	*B (SE)*	95% CI [LL, UL]	β	*p*
Gender	0.40 (0.15)	[0.11, 0.69]	.17	.007	0.09 (0.12)	[− 0.13, 0.32]	.05	.423	− 1.33 (0.43)	[− 2.19, − 0.48]	− .21	.002*
Age	0.20 (0.07)	[0.06, 0.34]	.17	.006	0.02 (0.06)	[− 0.09, 0.13]	.02	.723	− 0.35 (0.21)	[− 0.76, 0.06]	− .11	.096
Sexual experience	− 0.35 (0.19)	[− 0.72, 0.01]	− .12	.058	− 0.09 (0.14)	[− 0.38, 0.19]	− .04	.518	− 0.31 (0.54)	[− 1.38, 0.76]	− .04	.569
Sexual orientation	− 0.12 (0.14)	[− 0.39, 0.16]	− .05	.403	− 0.13 (0.11)	[− 0.34, 0.08]	− .08	.222	0.64 (0.41)	[− 0.16, 1.44]	.11	.117
Passive sexters^a^	− 0.86 (0.19)	[− 1.22, − 0.49]	− .29	< .001**	− 0.46 (0.14)	[− 0.74, − 0.18]	− .22	.002*	− 1.52 (0.54)	[− 2.59, − 0.45]	− .19	.006
Non-sexters^a^	− 1.24 (0.16)	[− 1.55, − 0.94]	− .52	< .001**	− 0.71 (0.12)	[− 0.95, − 0.48]	− .42	< .001**	− 1.45 (0.45)	[− 2.34, − 0.56]	− .23	.002*

Reference groups: Gender (male = 1), sexual experience (sexually experienced = 1), and sexual orientation (non-exclusively heterosexual = 1). ^a^ The reference group comprises active sexters. A Bonferroni-corrected alpha level was applied for multiple testing: .05/11 = .005. * *p* < .005. ** *p* < .001

From a normalcy perspective, we tested differences in indicators of sexual competence (see Table 5). Hypotheses 5 to 7 were not supported. Neither NS nor PS differed significantly from AS in sexual self-esteem, communication about sexuality, refusal assertiveness, or initiation assertiveness.

**Table 5:** Results of multiple linear regressions for indicators of sexual competence, aligned with the normalcy perspective, with sexting behavior (active sexters as reference group), gender, age, sexual experience (if applicable), and sexual orientation as predictors

	Sexual self-esteem (*n* = 213)				Communication about sexuality (*n* = 176)			
Predictor	*B (SE)*	95% CI [LL, UL]	β	*p*	*B (SE)*	95% CI [LL, UL]	β	*p*
Gender	0.30 (0.10)	[0.10, 0.50]	.20	.004*	0.15 (0.14)	[− 0.14, 0.43]	.08	.317
Age	0.11 (0.05)	[0.01, 0.20]	.15	.033	0.04 (0.07)	[− 0.10, 0.18]	.05	.560
Sexual experience	0.20 (0.13)	[− 0.05, 0.46]	.11	.113	–	–	–	–
Sexual orientation	− 0.09 (0.10)	[− 0.28, 0.10]	-.06	.367	0.32 (0.14)	[0.05, 0.58]	.19	.021
Passive sexters^a^	0.09 (0.13)	[− 0.16, 0.35]	.05	.461	0.26 (0.17)	[− 0.08, 0.59]	.12	.132
Non-sexters^a^	0.01 (0.11)	[− 0.20, 0.22]	.01	.946	0.17 (0.15)	[− 0.12, 0.46]	.09	.255

	Refusal assertiveness (*n* = 177)				Initiation assertiveness (*n* = 177)			
Predictor	*B (SE)*	95% CI [LL, UL]	β	*p*	*B (SE)*	95% CI [LL, UL]	β	*p*
Gender	− 0.15 (0.14)	[− 0.43, 0.13]	− .08	.301	0.10 (0.16)	[− 0.21, 0.41]	.05	.511
Age	0.01 (0.07)	[− 0.13, 0.14	.01	.902	0.05 (0.07)	[− 0.09, 0.20]	.06	.473
Sexual orientation	0.33 (0.13)	[0.07, 0.59]	.20	.014	0.03 (0.15)	[− 0.26, 0.31]	.02	.853
Passive sexters^a^	0.19 (0.17)	[− 0.14, 0.51]	.09	.254	− 0.13 (0.18)	[− 0.49, 0.22]	− .06	.459
Non-sexters^a^	0.34 (0.15)	[0.05, 0.63]	.18	.020	− 0.16 (0.16)	[− 0.47, 0.15]	− .08	.320

Reference groups: Gender (male = 1), sexual experience (sexually experienced = 1), and sexual orientation (non-exclusively heterosexual = 1). ^a^ The reference group comprises active sexters. A Bonferroni-corrected alpha level was applied (05/11 = .005). * *p* < .005. ** *p* < .001

### Follow-Up Analysis

While our grouping was based on the distinction between active and passive sexting proposed by Barrense-Dias et al. (2017), other research has considered asking others for a sext to be a form of active sexting engagement, recognizing the individual’s active role in initiating the exchange (e.g., Van Dijck et al., 2025). We therefore conducted analyses using an alternative grouping strategy, defining active sexters as individuals who had sent a sext and/or asked others for a sext, whereas exclusively passive sexters only received and/or had been asked for a sext. Results are reported in Tables SM4-SM6 in the supplementary material. Overall, the pattern of findings remained similar. The only deviation was that the previously significant difference in depression between AS and NS was no longer statistically significant under the alternative grouping.

## Discussion

Existing research has predominantly framed sexting as a problematic behavior, reflecting a deviancy perspective that emphasizes associated risks of sexting engagement (Döring, 2014). A growing normalcy discourse has challenged the risk-oriented framing by recognizing consensual sexting as a contemporary form of sexual expression and communication, pointing to potential positive aspects and benefits (Van Dijck et al., 2025). This study aimed to compare adolescents who voluntarily engaged in active sexting (i.e., individuals who had sent a sext of themselves or others at least rarely) with adolescents who were exclusively involved in passive sexting (i.e., individuals who received, asked, and/or had been asked for a sext at least rarely but had not sent a sext), and non-sexters (i.e., individuals with no involvement in either passive or active sexting) on a range of sexuality-related cognitions, sexual behaviors, and depression. Adding to the literature, we included variables related to the deviancy perspective and indicators of sexual competence, which are more closely aligned with the normalcy perspective on sexting.

### Prevalence of Sexting

The findings show a high prevalence of sexting among our sample of German adolescents. Overall, 66.2% of participants reported involvement in active or passive sexting, while 33.8% indicated no sexting experience. Half of the participants had voluntarily sent a sexually explicit image of themselves or others, reflecting active sexting engagement. Almost all active sexters also reported passive sexting. Additionally, 16.2% reported passive sexting only, meaning they had consensually received a sexually explicit image, had requested, or had been asked to send one without having sent one themselves. These prevalence rates, particularly for active sexting, are higher than those reported in previous meta-analyses and reviews (Barrense-Dias et al., 2017; Madigan et al., 2018; Mori et al., 2022). Nonetheless, some studies have found comparably high rates among adolescents. For example, Pistoni et al. (2023) found that 54.0% of their sample had engaged in sexting behaviors. Of these, 17.8% were actively involved in sexting, and 36.2% were only passively engaged. Another study of adolescents estimated that 54.8% of participants had sent a sext at least once, while 77.2% had received one (Morelli et al., 2017). However, prevalence rates between studies are difficult to compare due to differing conceptualizations and operationalizations of sexting. Furthermore, data collection for this study took place during the COVID-19 pandemic, when adolescents may have turned to sexting as an alternative for offline sexual behaviors that were not possible due to social distancing measures and lockdowns (Maes & Vandenbosch, 2022).

### Exploratory Group Comparisons in Sexting

In an exploratory analysis, we examined differences between the groups of non-sexting, exclusively passive sexting, and active sexting adolescents with regard to gender, sexual orientation, age, and sexual experience. No significant gender differences were found between the groups. Previous research on gender differences has yielded mixed results (Gámez-Guadix & de Santisteban, 2018; Ybarra & Mitchell, 2014). Similar to our results, other studies have not found significant differences in the sending or receiving of sexts between male and female participants (e.g., Clancy et al., 2021; Madigan et al., 2018). Yet, differences in sample characteristics, measurement approaches, and sexting definitions may contribute to the inconsistent findings regarding gender across studies.

Regarding sexual orientation, previous research has reported a higher prevalence of sexting among non-heterosexual individuals (e.g., Van Ouytsel et al., 2021). However, our findings did not reflect this pattern. In our sample, sexting was not significantly associated with sexual orientation; thus, adolescents identifying as non-exclusively heterosexual were not overrepresented in the groups characterized by active or passive sexting. However, reducing both the sexting measure and the sexual orientation variable to dichotomous categories may have limited variability and masked more nuanced associations within our sample. Further, we used the Kinsey scale (Kinsey et al., 1948) to specifically assess sexual orientation. However, sexual orientation during adolescence may still be developing and may not yet be fully understood. Following Van Ouytsel et al. (2021), asking about sexual attraction rather than sexual orientation might be a more developmentally appropriate approach for this age group.

With respect to age, no significant differences were found between the sexting groups. This contrasts with other studies that have identified a positive association between age and sexting (e.g., Gámez-Guadix & de Santisteban, 2018; Morelli et al., 2017). The increasing prevalence of sexting with age has been explained by sexual and relational development throughout adolescence (Madigan et al., 2018). In our sample, age was unrelated to sexual experience, which may partly explain the absence of age-related differences across the three sexting groups. However, the groups differed significantly in terms of sexual experience. Specifically, sexually experienced adolescents were overrepresented in the active sexting group, whereas the non-sexting group included more sexually inexperienced adolescents than expected. These findings align with previous studies indicating that sexting is associated with sexual behavior (e.g., Ybarra & Mitchell, 2014), suggesting that sexting may become part of adolescents’ sexual repertoire once they are sexually active.

### Evidence for the Deviancy Perspective

Results from multiple linear regressions provide overall support for the deviancy perspective, which posits that sexting is associated with higher levels of risk-related sexual behaviors and cognitions, as well as depressive symptoms. All hypotheses derived from the deviancy perspective were at least partially supported. Partly consistent with Hypothesis 1, adolescents who actively engaged in sexting reported significantly higher levels of risky sexual behavior than their non-sexting peers. However, no significant difference emerged between active sexters and exclusively passive sexters when applying the Bonferroni-corrected alpha level. The association between sexting and risky sexual behaviors has been documented in prior research (Kosenko et al., 2017; Mori et al., 2019). Actively sending a sexually explicit image, even voluntarily, entails the potential risk of unwanted dissemination. The observed associations between active sexting and risky sexual behaviors can therefore be understood within the theoretical framework of the problem behavior theory (PBT; Jessor, 2001). According to the PBT, adolescents’ problem or risk behaviors share common antecedents, such as greater attitudinal tolerance of deviance, and individuals who engage in one problem behavior are more prone to engage in others.

In line with Hypothesis 2, active sexters endorsed significantly riskier sexual scripts than both non-sexters and exclusively passive sexters. Furthermore, they reported significantly greater acceptance of sexual coercion compared to non-sexting adolescents. The findings suggest that active sexting is linked to behavioral risk-taking and to cognitive risk factors related to sexual aggression and risky sexual practices. Viewed through the lens of PBT, this pattern supports the notion of shared underlying individual factors, particularly beliefs and attitudes, that foster both active sexting and risk-related sexual behaviors and experiences. Further research is, however, needed to investigate the role of risk-related cognitions in the associations between sexting, sexual risk-taking, and coercive sexual behaviors, as indicated by previous research (Kosenko et al., 2017; Mori et al., 2019; Stanley et al., 2018).

Confirming aspects of Hypothesis 3, significant differences emerged between active sexters and both comparison groups in terms of pornography use and related perceptions. Non-violent pornography use was significantly higher among active sexters than non-sexters, and they also reported significantly more frequent violent pornography use than both non-sexters and exclusively passive sexters. As suggested by Clancy et al. (2021), pornography use may shape sexual scripts concerning self-representation and sexual behaviors, which are then replicated through sexting. Notably, in our study, active sexters also scored higher on perceived realism and utility of pornography than non-sexters and exclusively passive sexters, indicating a stronger tendency to regard pornographic content as realistic and useful for their own sexual interactions. A recent meta-analysis found a positive relationship between the frequency of pornography use and perceived realism of pornography (Gunnoo & Powell, 2023). This may explain why active sexters, who generally reported higher pornography use than non-sexters and more frequent consumption of violent content than exclusively passive sexters, also exhibited the highest levels of perceived realism. The combination of comparably high violent pornography use and elevated perceived realism among active sexters is noteworthy and warrants further investigation. Based on sexual script theory (Simon & Gagnon, 1986), depictions of aggressive and degrading behavior in sexual interactions may be incorporated in their sexual scripts and subsequently enacted in real-life encounters. Following social learning theory (Bandura, 1977), perceived realism of pornographic content increases the likelihood that observed behaviors are transferred into users’ own repertoire of sexual behaviors.

Finally, partly supporting Hypothesis 4, active sexters reported significantly higher levels of depressive symptoms than non-sexters, while the difference between active and exclusively passive sexters did not reach the adjusted significance threshold. Depressive symptoms may result from active sexting, particularly when images are distributed to third parties without consent (Sciacca et al., 2023). Moreover, unexpected or negative responses from the intended recipient may lead to feelings of rejection and perceived inadequacy. It is also possible that adolescents with depressive symptoms are more prone to actively engage in sexting to seek attention and validation from others (Gámez-Guadix & de Santisteban, 2018) in an attempt to address feelings of sadness and loneliness.

### Lack of Evidence for Potential Benefits

From a normalcy perspective, consensual sexting is regarded as a contemporary form of sexual expression and communication mediated by new technology (Döring, 2014). This approach acknowledges potential individual and relational benefits (Van Dijck et al., 2025). From this viewpoint, active sexting may manifest during normative sexual development and could be associated with indicators of sexual competence, such as higher sexual self-esteem, stronger sexual assertiveness, and more frequent communication about sexuality with a partner. We therefore hypothesized that active sexters would score higher on these indicators than non-sexters and exclusively passive sexters.

Overall, none of the hypotheses informed by the normalcy perspective (Hypotheses 5–7) were supported. First, active sexters did not report significantly higher sexual self-esteem than non-sexters or exclusively passive sexters (Hypothesis 5). By contrast, Marengo et al. (2019) found that adolescents who had sent a visual sext were more likely to have a more positive general sexual self-concept than those who had not. In their study, sexual self-concept was assessed using items referring to adolescents’ confidence and positive attitudes towards sex, including self-perceived sexual readiness and sexual self-efficacy. This construct potentially captured a broader construct than sexual self-esteem alone. Further research is needed to examine whether active sexting is associated with other positive cognitions regarding the evaluation of the sexual self. Second, active sexters did not report significantly more frequent communication about sexuality with a partner than non-sexting or exclusively passive sexting adolescents (Hypothesis 6). Similarly, Falconer et al. (2023) found no significant differences between sexters and non-sexters in the frequency of discussing sexual topics such as condom use and sexual history with a partner in adult relationships. Hence, sexting may represent a distinct form of sexual expression that does not necessarily co-occur with discussions about sexuality. Finally, no significant group differences emerged in initiation or refusal assertiveness (Hypothesis 7). Because the assertiveness subscales showed low internal consistency, the null finding should be interpreted cautiously. Future research using more reliable and developmentally sensitive measures is needed to clarify this potential relationship.

Contrary to our expectations, there were no significant differences in terms of sexual competence measures between active sexting adolescents and non-sexting or exclusively passive sexting adolescents. The absence of significant group differences does not necessarily refute the normalcy perspective. Instead, it might suggest that the potential developmental benefits of active sexting may become apparent over time or with repeated experience. Specifically, the frequency of active sexting may be more important than the sole sexting status. Potential benefits may unfold over time as adolescents gain more experience in relationships and sexting becomes more integrated in their sexual repertoire. For instance, a study among 200 female adolescents engaged in sexting found a significant positive association between sexting frequency and reported positive consequences, such as acceptance of one’s body and strength of the relationship (Bragard & Fisher, 2022). Furthermore, the relationship context of sexting could play a crucial role. Sexting within a trusted and stable relationship may potentially be linked to greater benefits in terms of sexual competence than sexting in casual or less emotionally connected interactions. However, this study did not address the relationship context of sexting, which requires further examination. Qualitative approaches to sexting represent another avenue for future research in which adolescents themselves can discuss the benefits they gain, or hope to gain, through sexting.

### Limitations

When interpreting the results, several limitations should be considered. First, our study relied on a convenience sample of adolescents aged 15 to 18 years from Germany. Moreover, female adolescents were overrepresented, and non-binary participants were excluded due to their small sample size. These factors limit the generalizability of results to a broader adolescent population. Future studies should aim to recruit random, representative samples of adolescents and include participants with diverse gender identities. Second, the relatively small sample size should be noted. Because this was a pilot study for a more extensive longitudinal research project, recruitment was limited by available funding and resources. However, our a priori power analysis indicated that the sample size was sufficient for testing the proposed hypotheses. Third, the cross-sectional design precludes conclusions about causal relationships or the directionality of effects between the study variables. Future longitudinal studies should examine the temporal sequences of sexting and its associated factors, including sexuality-related cognitions, as research in this area remains scarce. Fourth, regarding our sexting measure, active sexting was assessed using a single item. Although other studies have also used single questions to assess the sending of a sext (e.g., Pistoni et al., 2023; Temple & Choi, 2014), our item encompassed two behaviors: sending a sexually explicit image of oneself and sharing another person’s sext. While we intended to capture voluntary sexting engagement, the latter behavior could be regarded as non-consensual sexting. Specifically, even if an image is shared voluntarily, this may occur against the will or without the knowledge of the original creator or the depicted person(s). Such cases should be regarded as an aggressive and aggravated form of sexting (Wachs et al., 2021). Combining these behaviors into one group may have biased the results by merging potentially opposing effects, particularly regarding potential benefits. Future research should therefore clearly distinguish between consensual and non-consensual active sexting behavior. Finally, to test our hypotheses, we categorized adolescents into three groups based on their responses to the sexting items rather than using the mean scores for active and passive sexting as continuous predictors. However, higher frequencies of engagement might be associated with different risks and benefits than sexting that occurs only rarely, yet both were grouped in our study. Future research may therefore benefit from incorporating frequency-based sexting variables.

### Practical Implications

The present study found that most adolescents had experiences with passive sexting, and about half of the sample had engaged in sexting actively. These findings highlight the need to address adolescents’ sexting in educational programs, without reinforcing the moral panic that often surrounds the topic (Döring, 2014). Instead, parents, educators, and practitioners should discuss this topic in a non-stigmatizing manner that acknowledges its popularity among adolescents, while also addressing associated risks in an open dialogue. Adolescents need guidance on safe sexting practices, with particular attention to the role of consent, mutual respect, and privacy in digital exchanges. Given that active sexting was linked to a range of risk-related behaviors and sexuality-related cognitions, sexting should not be addressed in isolation but rather embedded within comprehensive sexual education programs aimed at mitigating potential risk factors and fostering sexual competence. Such programs could address related domains, such as sexual boundaries, consent, and pornography literacy.

#### Conclusion

The present study compared adolescents who actively engaged in voluntary sexting with those who had never or only passively engaged in sexting across sexual behaviors, sexuality-related cognitions, and depressive symptoms while considering both risk-related and positive indicators of sexual development. Consistent with the deviancy perspective, active sexters scored higher than non-sexters on all risk-related variables. Moreover, significant differences between exclusively passive and active sexters emerged in risky sexual scripts, perceived realism of pornography, and violent pornography use, further underscoring the potential risks associated with active sexting engagement. Contrary to our assumptions informed by the normalcy perspective, active sexters did not exhibit higher levels of the measured indicators of sexual competence than either exclusively passive sexters or non-sexters. The absence of significant group differences in sexual competence indicators suggests that potential developmental benefits of active sexting may depend on factors not examined in the current study, such as the relational context. Research that addresses both the risks and the potential benefits of adolescent sexting is warranted, particularly given the high prevalence of active sexting observed in this study. While some associations, such as those between sexting, risky sexual behavior, and depression, have been demonstrated in previous research, this study contributes to the literature by identifying differences in sexuality-related cognitions. In line with the PBT, the findings suggest that active sexting may be part of a broader constellation of risk behaviors driven by shared underlying factors. Incorporating sexual scripts theory into this line of reasoning, associations between sexting, risky sexual behavior, and sexual aggression may reflect shared cognitive representations that guide adolescents’ sexual decision-making and behavior.

## Supplementary Information

Below is the link to the electronic supplementary material.

Supplementary file1 (DOCX 56 kb)

## Data Availability

The data is available upon request.

## Footnotes

1 Although the sexting items aimed to capture voluntary behaviors, their wording may also encompass situations that were not voluntary or were non-consensual for other persons involved. With regard to the active sexting item, the potential inclusion of non-consensual forwarding is discussed in the Limitations section.

2 The German term “Geschlecht” refers to both sex and gender without explicitly differentiating between the concepts. Although the response options female (weiblich) and male (männlich) are regarded as sex categories (Canadian Institutes of Health Research, 2023), our wording is consistent with other German survey studies (de Vries et al., 2024). These terms have also been applied in the context of gender identity (e.g., Hadler et al., 2022). Nevertheless, future research should clearly distinguish between the two constructs by asking for biological sex and psychological gender identity in separate questions.

## Code Availability

The SPSS syntax is available upon request.
