# Supplementary material for: Active, Passive, and Non-Sexting Adolescents: Testing Deviancy and Normalcy Perspectives Across Risk-Related and Sexual Competence Variables
Source: Arch Sex Behav. 2026 Jul 1;55(5):2243–61. doi: 10.1007/s10508-026-03477-3 (PMC13427828; doi:10.1007/s10508-026-03477-3)
Supplement: Supplementary file 1 — Supplementary file1 (DOCX 56 kb) [file 10508_2026_3477_MOESM1_ESM.docx]

**Supplementary Material**

## **Active-, Passive-, and Non-Sexting Adolescents: Testing Deviancy and Normalcy Perspectives Across Risk-Related and Sexual Competence Variables**

## Additional analyses and alternative grouping strategies

Following the initial grouping strategy for active sexters (i.e., participants who had sent a sexually explicit image of themselves or others at least rarely), exclusively passive sexters (i.e., participants who had received, asked others for a sext, and/or had been asked for a sext at least rarely but had not sent one themselves), and non-sexters (i.e., participants who reported no engagement in either active or passive sexting), based on Barrense-Dias et al. (2017), we conducted additional regression analyses using an alternative dummy-coding scheme. In this alternative dummy-coding scheme, the non-sexter group served as a reference group, allowing for the examination of differences between exclusively passive sexters and non-sexters. Results are presented in Tables SM1 through SM3.

Further, we employed an alternative grouping strategy, classifying participants as active sexters when they reported an active role in sexting interactions at least rarely (i.e., sending and/or asking for a sexually explicit image), whereas exclusively passive sexters reported only passive involvement (i.e., receiving and/or being asked for a sexually explicit image at least rarely; Van Dijck et al., 2025). Using this alternative grouping strategy, 12.0% (*n* = 26) of the sample were categorized as exclusively passive sexters and 54.2% (*n* = 117) as active sexters. The proportion of non-sexters group remained at 33.8% (*n* = 73). Results of regression analyses adopting the alternative grouping with active sexters as the reference group are reported in Tables SM4 through SM6.

Table SM1

*Results of Multiple Linear Regressions for Sexuality-Related Cognitions, Depression, and Pornography Use, aligned with the Deviancy Perspective, with Sexting Behavior (Non-Sexters as Reference Group), Gender, Age, Sexual Experience, and Sexual Orientation as Predictors*

|  |  | Risky sexual scripts (*n* = 213) | | | |  | Acceptance of sexual coercion (*n* = 213) | | | |  | Non-violent pornography use (*n* = 213) | | | |
| --- | --- | --- | --- | --- | --- | --- | --- | --- | --- | --- | --- | --- | --- | --- | --- |
| Predictor |  | *B (SE)* | 95 % CI  [LL, UL] | *β* | *p* |  | *B (SE)* | 95 % CI  [LL, UL] | *β* | *p* |  | *B (SE)* | 95 % CI  [LL, UL] | *β* | *p* |
| Gender |  | -0.02 (0.33) | [-0.67, 0.63] | -.00 | .962 |  | 0.13 (0.14) | [-0.15, 0.41] | .06 | .356 |  | 1.47 (0.17) | [1.14, 1.80] | .52 | < .001** |
| Age |  | -0.17 (0.16) | [-0.48, 0.14] | -.07 | .282 |  | -0.01 (0.07) | [-0.14, 0.13] | -.01 | .920 |  | 0.19 (0.08) | [0.03, 0.35] | .14 | .019 |
| Sexual experience |  | 0.44 (0.41) | [-0.38, 1.25] | .07 | .289 |  | -0.05 (0.18) | [-0.40, 0.30] | -.02 | .770 |  | 0.06 (0.21) | [-0.36, 0.47] | .02 | .784 |
| Sexual orientation |  | -0.27 (0.31) | [-0.88, 0.34] | -.06 | .390 |  | -0.38 (0.13) | [-0.64, -0.12] | -.20 | .005 |  | 0.39 (0.16) | [0.08, 0.70] | .15 | .014 |
| Active Sexters^a^ |  | 2.31 (0.34) | [1.63, 2.99] | .49 | < .001** |  | 0.57 (0.15) | [0.28, 0.87] | .30 | < .001** |  | 0.75 (0.18) | [0.40, 1.09] | .29 | < .001** |
| Passive Sexters^a^ |  | 0.98 (0.46) | [0.08, 1.88] | .16 | .033 |  | 0.06 (0.20) | [-0.32, 0.45] | .03 | .745 |  | 0.45 (0.23) | [-0.01, 0.91] | .13 | .055 |
|  |  |  |  |  |  |  |  |  |  |  |  |  |  |  |  |
|  |  | Violent pornography use (*n* = 213) | | | |  | Perceived realism/utility of pornography (*n* = 213) | | | |  | Depression (*n* = 213) | | | |
| Predictor |  | *B (SE)* | 95 % CI  [LL, UL] | *β* | *p* |  | *B (SE)* | 95 % CI  [LL, UL] | *β* | *p* |  | *B (SE)* | 95 % CI  [LL, UL] | *β* | *p* |
| Gender |  | 0.40 (0.15) | [0.11, 0.69] | .17 | .007 |  | 0.09 (0.12) | [-0.13, 0.32] | .05 | .423 |  | -1.33 (0.43) | [-2.19, -0.48] | -.21 | .002* |
| Age |  | 0.20 (0.07) | [0.06, 0.34] | .17 | .006 |  | 0.02 (0.06) | [-0.09, 0.13] | .02 | .723 |  | -0.35 (0.21) | [-0.76, 0.06] | -.11 | .096 |
| Sexual experience |  | -0.35 (0.19) | [-0.72, 0.01] | -.12 | .058 |  | -0.09 (0.14) | [-0.38, 0.19] | -.04 | .518 |  | -0.31 (0.54) | [-1.38, 0.76] | -.04 | .569 |
| Sexual orientation |  | -0.12 (0.14) | [-0.39, 0.16] | -.05 | .403 |  | -0.13 (0.11) | [-0.34, 0.08] | -.08 | .222 |  | 0.64 (0.41) | [-0.16, 1.44] | .11 | .117 |
| Active Sexters^a^ |  | 1.24 (0.16) | [0.94, 1.55] | .56 | < .001** |  | 0.71 (0.12) | [0.48, 0.95] | .45 | < .001** |  | 1.45 (0.45) | [0.56, 2.34] | .25 | .002* |
| Passive Sexters^a^ |  | 0.38 (0.21) | [-0.02, 0.79] | .13 | .063 |  | 0.25 (0.16) | [-0.06, 0.57] | .12 | .112 |  | -0.08 (0.60) | [-1.26, 1.11] | -.01 | .900 |

*Note.* Reference groups: Gender (male = 1), sexual experience (sexually experienced = 1), and sexual orientation (non-exclusively heterosexual = 1). ^a^ The reference group comprises non-sexters. A Bonferroni-corrected alpha level was applied for multiple testing: .05/11 = .005. * *p* < .005. ** *p* < .001.

Table SM2

|  |  | Risky sexual behavior (*n* = 177) | | | |
| --- | --- | --- | --- | --- | --- |
| Predictor |  | *B (SE)* | 95 % CI [LL, UL] | *β* | *p* |
| Gender |  | -0.15 (0.10) | [-0.35, 0.04] | -.12 | .127 |
| Age |  | 0.03 (0.05) | [-0.06, 0.13] | .05 | .472 |
| Sexual orientation |  | 0.09 (0.09) | [-0.09, 0.28] | .08 | .315 |
| Active Sexters^a^ |  | 0.57 (0.10) | [0.37, 0.77] | .46 | < .001** |
| Passive Sexters^a^ |  | 0.30 (0.13) | [0.03, 0.56] | .19 | .027 |

*Results of Linear Regression Analysis for Risky Sexual Behavior as a Variable aligned with the Deviancy Perspective, with Sexting Behavior (Non-Sexters as Reference Group), Gender, Age, and Sexual Orientation as Predictors*

*Note.* Reference groups: Gender (male = 1), and sexual orientation (non-exclusively heterosexual = 1). ^a^ The reference group comprises non-sexters. Bonferroni-corrected alpha level was applied (05/11 = .005). * *p* < .005. ** *p* < .001.Table SM3

|  |  | Sexual self-esteem (*n* = 213) | | | |  | Communication about sexuality (*n* = 176) | | | |
| --- | --- | --- | --- | --- | --- | --- | --- | --- | --- | --- |
| Predictor |  | B (SE) | 95 % CI  [LL, UL] | *β* | *p* |  | B (SE) | 95 % CI  [LL, UL] | *β* | *p* |
| Gender |  | 0.30 (0.10) | [0.10, 0.50] | .20 | .004* |  | 0.15 (0.14) | [-0.14, 0.43] | .08 | .317 |
| Age |  | 0.11 (0.05) | [0.01, 0.20] | .15 | .033 |  | 0.04 (0.07) | [-0.10, 0.18] | .05 | .560 |
| Sexual experience |  | 0.20 (0.13) | [-0.05, 0.46] | .11 | .113 |  | - | - | - | - |
| Sexual orientation |  | -0.09 (0.10) | [-0.28, 0.10] | -.06 | .367 |  | 0.32 (0.14) | [0.05, 0.58] | .19 | .021 |
| Active Sexters^a^ |  | -0.01 (0.11) | [-0.22, 0.20] | -.01 | .946 |  | -0.17 (0.15) | [-0.46, 0.12] | -.10 | .255 |
| Passive Sexters^a^ |  | 0.09 (0.14) | [-0.19, 0.37] | .05 | .538 |  | 0.09 (0.19) | [-0.30, 0.47] | .04 | .652 |
|  |  |  |  |  |  |  |  |  |  |  |
|  |  | Refusal assertiveness (*n* = 177) | | | |  | Initiation assertiveness (*n* = 177) | | | |
| Predictor |  | B (SE) | 95 % CI  [LL, UL] | *β* | *p* |  | B (SE) | 95 % CI [LL, UL] | *β* | *p* |
| Gender |  | -0.15 (0.14) | [-0.43, 0.13] | -.08 | .301 |  | 0.10 (0.16) | [-0.21, 0.41] | .05 | .511 |
| Age |  | 0.01 (0.07) | [-0.13, 0.14 | .01 | .902 |  | 0.05 (0.07) | [-0.09, 0.20] | .06 | .473 |
| Sexual orientation |  | 0.33 (0.13) | [0.07, 0.59] | .20 | .014 |  | 0.03 (0.15) | [-0.26, 0.31] | .02 | .853 |
| Active Sexters^a^ |  | -0.34 (0.15) | [-0.63, -0.05] | -.21 | .020 |  | 0.16 (0.16) | [-0.15, 0.47] | .09 | .320 |
| Passive Sexters^a^ |  | -0.15 (0.19) | [-0.53, 0.22] | -.07 | .428 |  | 0.02 (0.21) | [-0.39, 0.43] | .01 | .907 |

*Results of Multiple Linear Regressions for Indicators of Sexual Competence, aligned with the Normalcy Perspective, with Sexting Behavior (Non-Sexters as Reference Group), Gender, Age, Sexual Experience (if applicable), and Sexual Orientation as Predictors*

*Note.* Reference groups: Gender (male = 1), sexual experience (sexually experienced = 1), and sexual orientation (non-exclusively heterosexual = 1). ^a^ The reference group comprises non-sexters. Bonferroni-corrected alpha level was applied (05/11 = .005). * *p* < .005. ** *p* < .001.

Table SM4

|  |  | Risky sexual scripts (*n* = 213) | | | |  | Acceptance of sexual coercion (*n* = 213) | | | |  | Non-violent pornography use (*n* = 213) | | | |
| --- | --- | --- | --- | --- | --- | --- | --- | --- | --- | --- | --- | --- | --- | --- | --- |
| Predictor |  | *B (SE)* | 95 % CI  [LL, UL] | *β* | *p* |  | *B (SE)* | 95 % CI  [LL, UL] | *β* | *p* |  | *B (SE)* | 95 % CI  [LL, UL] | *β* | *p* |
| Gender |  | -0.14 (0.33) | [-0.79, 0.52] | -.03 | .681 |  | 0.09 (0.14) | [-0.19, 0.37] | .04 | .533 |  | 1.45 (0.17) | [1.12, 1.78] | .51 | < .001** |
| Age |  | -0.13 (0.16) | [-0.44, 0.18] | -.05 | .406 |  | 0.01 (0.07) | [-0.12, 0.15] | .01 | .877 |  | 0.20 (0.08) | [0.04, 0.36] | .14 | .013 |
| Sexual experience |  | 0.39 (0.42) | [-0.43, 1.20] | .06 | .353 |  | -0.07 (0.18) | [-0.42, 0.28] | -.03 | .700 |  | 0.05 (0.21) | [-0.37, 0.46] | .01 | .822 |
| Sexual orientation |  | -0.23 (0.31) | [-0.84, 0.39] | -.05 | .465 |  | -0.37 (0.14) | [-0.64, -0.11] | -.19 | .006 |  | 0.40 (0.16) | [0.09, 0.71] | .15 | .013 |
| Passive Sexters^a^ |  | -1.37 (0.46) | [-2.29, -0.46] | -.19 | .003* |  | -0.43 (0.20) | [-0.83, -0.04] | -.15 | .032 |  | -0.26 (0.24) | [-0.72, 0.20] | -.07 | .272 |
| Non-Sexters^a^ |  | -2.25 (0.34) | [-2.93, -1.58] | -.45 | < .001** |  | -0.54 (0.15) | [-0.83, -0.24] | -.26 | < .001** |  | -0.72 (0.17) | [-1.07, -0.38] | -.26 | < .001** |
|  |  |  |  |  |  |  |  |  |  |  |  |  |  |  |  |
|  |  | Violent pornography use (*n* = 213) | | | |  | Perceived realism/utility of pornography (*n* = 213) | | | |  | Depression (*n* = 213) | | | |
| Predictor |  | *B (SE)* | 95 % CI  [LL, UL] | *β* | *p* |  | *B (SE)* | 95 % CI  [LL, UL] | *β* | *p* |  | *B (SE)* | 95 % CI  [LL, UL] | *β* | *p* |
| Gender |  | 0.32 (0.15) | [0.03, 0.62] | .13 | .032 |  | 0.05 (0.12) | [-0.18, 0.28] | .03 | .669 |  | -1.43 (0.44) | [-2.30, -0.56] | -.23 | .001* |
| Age |  | 0.23 (0.07) | [0.09, 0.37] | .19 | .002* |  | 0.03 (0.06) | [-0.08, 0.14] | .04 | .550 |  | -0.29 (0.21) | [-0.70, 0.13] | -.09 | .176 |
| Sexual experience |  | -0.39 (0.19) | [-0.76, -0.02] | -.13 | .041 |  | -0.11(0.14) | [-0.40, 0.17] | -.05 | .437 |  | -0.34 (0.55) | [-1.43, 0.75] | -.04 | .534 |
| Sexual orientation |  | -0.09 (0.14) | [-0.37, 0.18] | -.04 | .506 |  | -0.12 (0.11) | [-0.33, 0.10] | -.07 | .281 |  | 0.63 (0.42) | [-0.19, 1.45] | .11 | .129 |
| Passive Sexters^a^ |  | -0.85 (0.21) | [-1.27, -0.44] | -.25 | < .001** |  | -0.50 (0.16) | [-0.81, -0.18] | -.21 | .002* |  | -0.79 (0.62) | [-2.00, 0.43] | -.09 | .204 |
| Non-Sexters^a^ |  | -1.20 (0.16) | [-1.51, -0.89] | -.51 | < .001** |  | -0.70 (0.12) | [-0.93, -0.46] | -.42 | < .001** |  | -1.24 (0.46) | [-2.13, -0.34] | -.20 | .007 |

*Results of Multiple Linear Regressions for Sexuality-Related Cognitions, Depression, and Pornography Use, aligned with the Deviancy Perspective,* *with Sexting Behavior (Active Sexters as Reference Group), Gender, Age, Sexual Experience, and Sexual Orientation as Predictors, using an Alternative Grouping Strategy for Active Sexters (i.e., Sending and/or Asking for a Sext), and Passive Sexters (i.e., Receiving and/or Being Asked for a Sext)*

*Note.* Reference groups: Gender (male = 1), sexual experience (sexually experienced = 1), and sexual orientation (non-exclusively heterosexual = 1). ^a^ The reference group comprises participants who asked for a sext and/or sent a sext at least rarely (i.e., active sexters). A Bonferroni-corrected alpha level was applied for multiple testing: .05/11 = .005. * *p* < .005. ** *p* < .001.

Table SM5

|  |  | Risky sexual behavior (*n* = 177) | | | |
| --- | --- | --- | --- | --- | --- |
| Predictor |  | *B (SE)* | 95 % CI [LL, UL] | *β* | *p* |
| Gender |  | -0.18 (0.10) | [-0.38, 0.02] | -.14 | .072 |
| Age |  | 0.05 (0.05) | [-0.05, 0.14] | .07 | .338 |
| Sexual orientation |  | 0.10 (0.10) | [-0.09, 0.28] | .08 | .305 |
| Passive Sexters^a^ |  | -0.23 (0.13) | [-0.49, 0.03] | -.13 | .082 |
| Non-Sexters^a^ |  | -0.55 (0.10) | [-0.75, -0.35] | -.39 | < .001** |

*Results of Linear Regression Analysis for Risky Sexual Behavior as a Variable aligned with the Deviancy Perspective,* *with Sexting Behavior (Active Sexters as Reference Group), Gender, Age, and Sexual Orientation as Predictors, using an Alternative Grouping Strategy for Active Sexters (i.e., Sending and/or Asking for a Sext), and Passive Sexters (i.e., Receiving and/or Being Asked for a Sext)*

*Note.* Reference groups: Gender (male = 1), and sexual orientation (non-exclusively heterosexual = 1). ^a^ The reference group comprises participants who asked for a sext and/or sent a sext at least rarely (i.e., active sexters). Bonferroni-corrected alpha level was applied (05/11 = .005). * *p* < .005. ** *p* < .001.

Table SM6

|  |  | Sexual self-esteem (*n* = 213) | | | |  | Communication about sexuality (*n* = 176) | | | |
| --- | --- | --- | --- | --- | --- | --- | --- | --- | --- | --- |
| Predictor |  | B (SE) | 95 % CI  [LL, UL] | *β* | *p* |  | B (SE) | 95 % CI  [LL, UL] | *β* | *p* |
| Gender |  | 0.30 (0.10) | [0.10, 0.50] | .21 | .003* |  | 0.18 (0.14) | [-0.11, 0.46] | .10 | .226 |
| Age |  | 0.10 (0.05) | [0.01, 0.20] | .14 | .039 |  | 0.03 (0.07) | [-0.10, 0.17] | .04 | .637 |
| Sexual experience |  | 0.21 (0.13) | [-0.05, 0.46] | .12 | .110 |  | - | - | - | - |
| Sexual orientation |  | -0.09 (0.10) | [-0.27, 0.10] | -.06 | .377 |  | 0.31 (0.14) | [0.04, 0.57] | .18 | .025 |
| Passive Sexters^a^ |  | 0.04 (0.14) | [-0.25, 0.32] | .02 | .803 |  | 0.32 (0.19) | [-0.06, 0.69] | .13 | .095 |
| Non-Sexters^a^ |  | -0.01 (0.11) | [-0.22, 0.20] | -.01 | .937 |  | 0.16 (0.15) | [-0.12, 0.45] | .09 | .259 |
|  |  |  |  |  |  |  |  |  |  |  |
|  |  | Refusal assertiveness (*n* = 177) | | | |  | Initiation assertiveness (*n* = 177) | | | |
| Predictor |  | B (SE) | 95 % CI  [LL, UL] | *β* | *p* |  | B (SE) | 95 % CI [LL, UL] | *β* | *p* |
| Gender |  | -0.13 (0.14) | [-0.41, 0.16] | -.07 | .377 |  | 0.08 (0.16) | [-0.22, 0.39] | .04 | .590 |
| Age |  | 0.00 (0.07) | [-0.13, 0.14] | .00 | .968 |  | 0.05 (0.07) | [-0.09, 0.20] | .06 | .460 |
| Sexual orientation |  | 0.32 (0.13) | [0.06, 0.59] | .19 | .016 |  | 0.04 (0.15) | [-0.25, 0.33] | .02 | .780 |
| Passive Sexters^a^ |  | 0.23 (0.19) | [-0.13, 0.60] | .10 | .207 |  | -0.27 (0.20) | [-0.67, 0.12] | -.11 | .176 |
| Non-Sexters^a^ |  | 0.34 (0.14) | [0.06, 0.62] | .18 | .020 |  | -0.17 (0.16) | [-0.48, 0.13] | -.09 | .267 |

*Results of Multiple Linear Regressions for Indicators of Sexual Competence, aligned with the Normalcy Perspective, with Sexting Behavior (Active Sexters as Reference Group), Gender, Age, Sexual Experience, and Sexual Orientation as Predictors, using an Alternative Grouping Strategy for Active Sexters (i.e., Sending and/or Asking for a Sext), and Passive Sexters (i.e., Receiving and/or Being Asked for a Sext)*

*Note.* Reference groups: Gender (male = 1), sexual experience (sexually experienced = 1), and sexual orientation (non-exclusively heterosexual = 1). ^a^ The reference group comprises participants who asked for a sext and/or sent a sext at least rarely (i.e., active sexters). Bonferroni-corrected alpha level was applied (05/11 = .005). * *p* < .005. ** *p* < .001.

**References**

Barrense-Dias, Y., Berchtold, A., Suris, J. C., & Akre, C. (2017). Sexting and the definition issue. *Journal of Adolescent Health*, *61*(5), 544-554. <https://doi.org/10.1016/j.jadohealth.2017.05.009>

Van Dijck, S., Van den Eynde, S., & Enzlin, P. (2025). The bright side of sexting: A scoping review on its benefits. *Computers in Human Behavior*, *164*, 108499. <https://doi.org/10.1016/j.chb.2024.108499>
